# Hepatitis C: evaluation of outcomes and georeferencing of cases in Santa Cruz do Sul, Brazil, between 2002 and 2015. A cross-sectional study

**DOI:** 10.1590/1516-3180.2017.0169180917

**Published:** 2017-12-04

**Authors:** Lia Goncalves Possuelo, Daiane Perin, Patricia Faber Breunig, Daniel Felipe Schroeder, Manuela Filter Allgayer, Camilo Darsie, Marcelo Carneiro, Vanda Hermes, Jane Dagmar Pollo Renner

**Affiliations:** I MSc, PhD. Researcher, Department of Biology and Pharmacy, Universidade de Santa Cruz do Sul (UNISC), Santa Cruz do Sul (RS), Brazil.; II BSc. Pharmacist, Department of Biology and Pharmacy, Universidade de Santa Cruz do Sul (UNISC), Santa Cruz do Sul (RS), Brazil.; III Student. Pharmacist, Department of Biology and Pharmacy, Universidade de Santa Cruz do Sul (UNISC), Santa Cruz do Sul (RS), Brazil.; IV BSc. Geographer, Department of History and Geography, Universidade de Santa Cruz do Sul (UNISC), Santa Cruz do Sul (RS), Brazil.; V MSc. Nurse and Postgraduate Student, Universidade de Santa Cruz do Sul (UNISC), Santa Cruz do Sul (RS), Brazil.; VI PhD. Professor, Department of History and Geography, Universidade de Santa Cruz do Sul (UNISC), Santa Cruz do Sul (RS), Brazil.; VII PhD. Professor, Department of Biology and Pharmacy, Universidade de Santa Cruz do Sul (UNISC), Santa Cruz do Sul (RS), Brazil.; VIII Specialist. Nurse, Department of Health of Santa Cruz do Sul, Santa Cruz do Sul (RS), Brazil.; IX MSc, PhD. Researcher, Department of Biology and Pharmacy, Universidade de Santa Cruz do Sul (UNISC), Santa Cruz do Sul (RS), Brazil.

**Keywords:** Hepatitis C, Genotype, Prevalence, Death

## Abstract

**BACKGROUND:**

Hepatitis C virus infection is one of the main causes of chronic liver disease, with high death rates. The aim here was to analyze case outcomes, sociodemographic and clinical characteristics and spatial distribution among patients diagnosed with hepatitis C in the city of Santa Cruz do Sul (RS), Brazil.

**DESIGN AND SETTING:**

Cross-sectional study on 200 cases of hepatitis C in Santa Cruz do Sul that were notified between 2002 and 2015.

**METHODS:**

Secondary data including sociodemographic and clinical variables and type of outcome (death, follow-up, abandonment or clinical cure) were gathered. The spatial distribution analysis on hepatitis C virus cases according to outcome was based on information regarding residential address.

**RESULTS:**

58.5% of the patients were 41 years of age and over, 67% were males and 92.5% had the chronic form of the disease. The most frequent transmission route was illicit drug injection (29%); 15.1% of the patients presented coinfection with the human immunodeficiency virus (HIV). Regarding outcomes, 31% achieved clinical cure, 10% died and 20% abandoned follow-up. The cases studied were mainly located in regions of the city characterized by lower socioeconomic status, with high frequency of places used for drug trafficking.

**CONCLUSION:**

The population consisted of adults aged 41 years and over, mostly with chronic hepatitis C. The most common transmission routes were illicit drug injection and blood transfusions. There were high rates of HIV coinfection and abandonment of disease monitoring and predominance of cases in neighborhoods with low socioeconomic status.

## INTRODUCTION

The hepatitis C virus (HCV) is one of the leading causes of chronic liver disease worldwide and is characterized as a silent disease that is difficult to identify in most patients.^1^^,^^2^ Approximately 15%-45% of infected individuals spontaneously clear the virus within six months of infection without any treatment, and the remaining 55%-85% of them may progress to persistent chronic infection. It has been estimated that the risk of developing cirrhosis within 20 years is 15%-30% in those with chronic HCV infection, and the risk of developing hepatocellular carcinoma is 1%-4% per year.^3^^,^^4^ At a global level, around 71 million people have HCV, with approximately 399,000 deaths annually.^5^

In Brazil, the data show that the numbers of deaths due to HCV have been increasing over time in all regions. Between 2000 and 2015, 46,314 deaths related to HCV were identified.^1^ The southern region presented a coefficient of mortality due to HCV as the underlying cause of death for 1.5/100,000 inhabitants. In the state of Rio Grande do Sul (RS), this coefficient was 2.8/100,000 inhabitants, which was almost three times larger than the national mean for the same period.^1^ The data on the extent and spatial distribution of HCV outcomes in the city of Santa Cruz do Sul (RS) is limited, which thus highlights the usefulness of the present study.

The main route for HCV transmission is the parenteral route, through direct or percutaneous contact with contaminated blood. Additionally, it is also transmitted through sexual activity and the vertical route.^1^^,^^2^^,^^3^ The groups at greatest risk of contracting HCV are illicit drug users, individuals with piercings and tattoos that were made using contaminated tools, HIV-positive patients, individuals who received blood before 1992, healthcare professionals and patients undergoing hemodialysis.^1^^,^^2^^,^^3^ Other less common forms of contamination are the parenteral route, which may occur through medical, dental, manicure and acupuncture procedures; and sharing of objects within the household.^6^^,^^7^

HCV prevention and control depends on a complex assessment of HCV infection, which involves correlation of risk factors and estimation of factors that accelerate disease progression.^8^^,^^9^ Because there is no vaccine for HCV, or any type of post-exposure prophylaxis, proper epidemiological evaluation is essential for planning primary HCV prevention in any population. Follow-up of HCV-positive individuals by the healthcare team is of utmost importance, rather than sporadic medical evaluation. Such follow-up is required in order to establish what the treatment should be and to ensure adherence to treatment. HCV treatment is available free of charge through the Brazilian National Health System (SUS).^10^

In the light of this situation, the objective of the present study was to analyze case outcomes, sociodemographic and clinical characteristics and spatial distribution among HCV cases that were notified between 2002 and 2015 in the city of Santa Cruz do Sul (RS), Brazil.

## METHODS

A cross-sectional study was carried out, in which all HCV cases diagnosed and notified in the urban area of the municipality of Santa Cruz do Sul (RS), Brazil, between 2002 and 2015, were included. HCV infection was defined as the presence of HCV ribonucleic acid (HCV-RNA) and anti-HCV antibodies in serum or plasma.^1^ A positive HCV antibody test was taken to indicate exposure to HCV, which could represent current or past infection. A positive HCV-RNA test indicated current HCV infection.^11^ Santa Cruz do Sul is located in the central region of the state of Rio Grande do Sul, at a distance of 155 km from the state capital, Porto Alegre. The estimated population of Santa Cruz do Sul is 127,516 inhabitants and its human development index is 0.773.^12^

Secondary data collection was carried out from March 2016 to August 2017, at the viral hepatitis reference unit of Santa Cruz do Sul (RS). The data were collected from the Notifiable Diseases Information System (SINAN), Mortality Information System (SIM) and Laboratory Environment System (GAL) of the Ministry of Health and from electronic records (Fly Saúde) of the Municipal Health Department of Santa Cruz do Sul.

The data collection tool included the following sociodemographic variables: gender, age, ethnicity, schooling level and occupation; and clinical variables: associated diseases, clinical form of the disease, probable source of infection and HCV genotype. These data were collected from SINAN. Data relating to case outcome (death, follow-up, abandonment or clinical cure) were collected from SIM, electronic records and GAL. Situations in which patients had not come to their medical appointments for the last six months were considered to constitute abandonment of treatment. Situations in which patients had undetectable levels of HCV-RNA four weeks after treatment were considered to constitute clinical cure. Patients whose cases had been notified to SINAN but for whom there were no data in electronic records, SIM or GAL were considered to be unlocatable.

The spatial distribution of HCV cases was determined based on information regarding the residential address found in SINAN. The distribution analysis on the cases was performed using the representation technique, by counting points according to the case outcome. The graphical representation of the mapping was produced using the Quantum GIS 2.14.3-Essen (QGIS) software. To create thematic maps, the Google Earth Pro software, which is available free of charge at https://www.google.com.br/earth/download/gep/agree.html and the Quantum GIS 2.14.3-Essen (QGIS) software, which is also available free of charge at https://www.qgis.org/en/site/forusers/download.html, were used. The geoprocessing sector of the municipality of Santa Cruz do Sul, at http://www.santacruz.rs.gov.br/geo/, provided free vector base maps of the urban area of the municipality, along with vector maps of the streets and avenues, districts and urban perimeter. These data were entered into the QGIS software, which was used to produce all thematic and spatial distribution editions of the maps. The outcomes from the HCV cases were georeferenced using Google Earth Pro software and were then exported in kml (Keyhole Markup Language) format. These points were inserted as an overlay in the QGIS, so that they could be located. The final layouts of the maps were also produced using the QGIS software.

The data were input and analyzed using the SPSS software, version 23.0. A descriptive analysis was carried out, which included investigation of the frequency distribution of sociodemographic, clinical and outcome variables. The data were presented as absolute numbers, frequencies and/or means.

This project was approved by the Research Ethics Committee of Universidade de Santa Cruz do Sul (UNISC), under number 1,361,022.

## RESULTS

A total of 200 cases were analyzed. Sociodemographic, clinical and outcome data are shown in Table 1. Regarding age, 58.5% of the patients were aged 41 years or older, with a range from 13 to 75 years. There were 134 male cases (67%).

**Table 1:** Sociodemographic, clinical and outcome characterization of the population with hepatitis C virus in the municipality of Santa Cruz do Sul (RS), 2002-2015

Variable	n = 200	%
Sex		
Male	134	67
Female	66	33
Age (years)		
13-20	3	1.5
21-30	26	13
31-40	54	27
41-50	60	30
> 50	57	28.5
Ethnicity		
Caucasian	164	82
Non-Caucasian	36	18
Level of schooling		
Illiterate	2	1
Elementary school (incomplete/complete)	164	67
High school (incomplete/complete)	134	32
Occupation		
Retired	49	24.5
Driver	17	8.5
Factory worker*	38	19
Teacher	11	5.5
Mason	24	12
Farmer	5	2.5
Soccer player	3	1.5
Prison inmate	12	6
Beauty professional^†^	14	7
Unknown	27	13.5
In prison		
Yes	12	6
No	188	94
Associated diseases		
HIV/AIDS	30	15.1
HBV	2	1
HAV	1	0.5
Clinical form		
Acute hepatitis	15	7.5
Chronic hepatitis	185	92.5
Probable source of infection		
Sexual	9	4.5
Transfusion	25	12.5
Illicit drug use	58	29.0
At home	1	0.5
Surgical treatment	6	3.0
Unknown	101	50.5
HCV genotype		
Genotype 1	104	52.0
Genotype 2	10	5.0
Genotype 3	58	28.5
Unknown	28	14.5
Outcome		
Clinical cure	62	31.0
Undergoing follow-up	45	22.5
Unlocatable	33	16.5
Abandonment of treatment	40	20.0
Death	20	10.0

HCV = hepatitis C virus; HIV/AIDS = human immunodeficiency virus/acquired immunodeficiency syndrome; HBV = hepatitis B virus; HAV = hepatitis A virus; *tobacco industry worker; ^†^manicurist, hairdresser, beautician.

Thirty patients (15.1%) were coinfected with HCV and HIV and, among these patients, eight (26.7%) had abandoned their treatments. The chronic form of HCV was identified in 185 patients (92.5%). Regarding the sources of infection, the most frequent of these, reported by individuals evaluated here, were illicit drug use (29%) and blood transfusions (12.5%) (Table 1). Twelve patients (6%) were institutionalized in a prison when their cases were notified, and five of these (41.7%) subsequently abandoned their treatments.

Death was reported in a total of 20 cases (10%). HCV was the underlying cause of death in nine cases (45%), HIV in three (15%) and other causes in eight (40%). A total of 73 patients (36.5%) were classified as either having abandoned their treatments or being unlocatable. Among these, 16 (21.9%) were coinfected with HCV and HIV.

**Figure 1:**
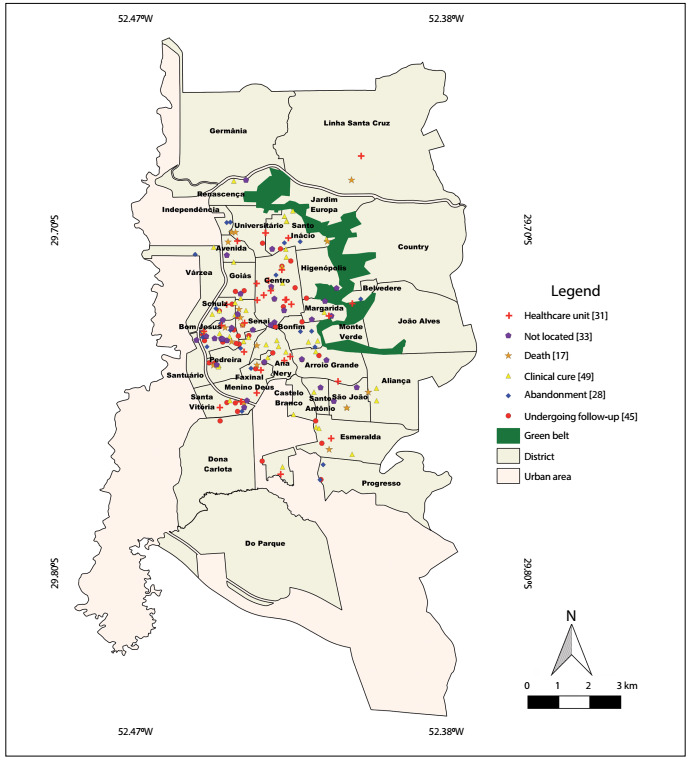
Map of the municipality of Santa Cruz do Sul (RS) showing the spatial distribution of hepatitis C virus cases, classified according to the genotype.

It was possible to describe the spatial distribution of HCV cases in relation to 172 patients (86%). The numbers of HCV cases were higher in regions of the municipality with lower socioeconomic status and high frequency of places used for drug trafficking, and in the regional prison (Figure 1).

## DISCUSSION

This study population predominantly comprised adult individuals aged 41 years and over. The reported transmission routes were, most frequently, illicit drugs and blood transfusions. Among all the cases, 92.5% had chronic hepatitis, 52% had genotype 1, 10% died, 31% achieved clinical cure and 15.1% were coinfected with HIV.

In a study carried out among HCV-positive individuals who were attended at a public service in Porto Alegre, Brazil, it was found that the mean age of the individuals assessed was 40.1 years.^13^ Other studies have also shown higher numbers of cases among individuals older than 40 years of age.^13^^,^^14^ Similar results were observed in our study. The diagnosis of hepatitis C is more frequent in adulthood and among the elderly, given that it is a silent and chronic disease, and because these individuals may have undergone some type of surgery with inadequately sterilized instruments and unsafe blood transfusions before the year 1993, when there was no screening for hepatitis C in blood banks yet.^14^^,^^15^

Regarding sex, the hepatitis C cases were predominant among male individuals (67%), thus corroborating other studies.^13^^,^^16^ In Brazil, 106,637 HCV cases occurred among males between 1999 and 2015, representing 58.5% of all cases.^1^ The higher prevalence of hepatitis C virus infection among males can be explained by their exposure to risk factors, or by the fact that the diagnosis of HCV is made when men are blood donation volunteers.^15^ HCV infection affects both men and women, but there are no comprehensive studies confirming that men are more vulnerable to this infection.^15^

The main routes of HCV transmission have been found to be illicit drugs, blood transfusions (mainly before 1992), sharing of materials for drug use, lack of personal hygiene and presence of tattoos and piercings.^17^^,^^18^ The risk of sexual transmission is low, but recent data have shown that promiscuous male homosexual activity is associated with HCV infection.^18^ In the present study, 29% of the patients reported drug use and 12.5% had had blood transfusions, but the year of the blood transfusion was not mentioned. These results agree with data from the Brazilian Ministry of Health, in which it was reported that use of drugs was the main route for HCV transmission among the cases notified (29.2%).^1^ In another study, conducted in Palhoça, state of Santa Catarina (SC), which was based on HCV notifications in the years 2008 to 2010, the risk factors most often involved in disease transmission were use of illicit injection drugs (34.5%), use of inhalable drugs and crack (33.5%) and blood transfusions (28.6%).^14^

Coinfection with HIV is common among hepatitis C carriers, since these viruses have similar transmission routes, mainly through the parenteral route.^19^ In the present study, 15.1% of the individuals had HCV/HIV coinfection. About 2.3 million people living with HIV around the world are coinfected with HCV.^20^ The HCV/HIV coinfection rate for Brazil between 2007 and 2016 was 9.8% and it varied according to the region.^1^ The southern region had the highest coinfection rate in Brazil (13.3%), and this was similar to what was observed in the present study. In cases in which coinfection occurs, the host’s immune response is impaired and the prognosis for both infections is worsened: liver impairment is accelerated, with progression to cirrhosis, liver failure and liver carcinoma, which can lead to the death of the infected individual.^19^^,^^21^ Antiretroviral therapy, which improves the survival of HIV-infected patients, may contribute towards liver injury and lead to hepatotoxicity in cases of coinfection, thereby accentuating the liver injury caused by the hepatitis virus. The risks of hepatotoxicity in coinfected patients are three to four times higher than in non-infected patients, due to liver disorders.^21^

Regarding the form of the disease, 92.5% of the individuals in the present study had the chronic form and 10% of them died. A systematic review of the literature suggested that approximately 130-150 million people are living with chronic hepatitis C.^22^ In Brazil, between 1999 and 2016, more than 155,000 cases of chronic HCV cases were confirmed.^1^ Between 1990 and 2013, the number of deaths worldwide due to viral hepatitis increased from 0.89 million to 1.45 million.^23^

In the absence of treatment, 20-30% of hepatitis B virus (HBV) and HCV-infected individuals will develop hepatocellular carcinoma or cirrhosis. It has been estimated that this will lead to 19 million deaths between 2015 and 2030 (11.8 million due to HBV and 7.2 million due to HCV) around the world.^23^ In the present study, 22 cases (11%) developed cirrhosis or hepatocellular cancer.

Treatment for hepatitis is available free of charge through the Brazilian public health system (SUS) and has the purpose of reducing the risk of developing cirrhosis and liver carcinoma. The most widely used pharmacological treatment for HCV was for many years based on a combination of interferon and ribavirin.^24^ Treatment regimens based on interferon may be problematic because of the high frequency of adverse effects and the inconvenience of weekly injections. Thus, their effectiveness may be limited especially among patients whose clinical condition is more severe.^24^^,^^25^

Recently, the introduction of therapeutic regimens including new drugs such as sofosbuvir and daclatasvir has shown positive results regarding HCV treatment.^26^ Among the patients analyzed here, it was seen that 31% presented clinical cure, regardless of the therapeutic regimen used. Recently identified symptomatic or non-symptomatic acute infection is an important HCV control measurement. Late treatment onset is associated with a worse sustained virological response (SVR). A situation of SVR consists of non-detection of HCV-RNA 12 or 24 weeks after treatment. If the infection has been recently treated, SVR rates may reach values greater than 80%.^27^

One limitation of this study was indeed the impossibility of evaluating the therapeutic regimens used individually. A total of 33% of the patients were considered to be unlocatable in any of the public information systems investigated. Some of these patients may have sought treatment within the private sector.

Among the patients of the present study, 45 (22.5%) are still being followed up, and 40 (20%) abandoned their treatment. HCV is a disease that requires discipline among patients and understanding from healthcare professionals, and situations that can significantly interfere with the success of follow-up and treatment adherence need to be identified rapidly.^27^ Thus, it is crucial that healthcare professionals should establish a solid relationship with their patients.^10^^,^^21^ To establish proper treatment for patients with chronic HCV, it is extremely important that their medical records, anamnesis and physical examination should be rigorously completed and that they are included within the routine of screening and referral services. The same is true regarding filling out forms that are used in disease notification and requests for examinations. Adherence to healthcare services among patients with chronic hepatitis C is essential, for the healthcare strategy to be successful.^27^

During the pretreatment period, it is also necessary to make a careful evaluation of patients’ comorbidities and coinfections caused by HBV and HIV, along with their clinical, psychiatric and social conditions.^21^^,^^28^ Because of the pathophysiological characteristics of chronic HCV infection, at least four consultations per year are required. This routine should be individualized among patients for whom therapy was recently started or in situations in which the risk of adverse events requires priority care.^21^

Genotype 1 (52%) was most prevalent in the present study, which confirms that in RS and Brazil, genotype 1 is the one most frequently identified. This is also the genotype that is the most resistant and the most difficult to treat.^14^ HCV genotype 1 is most prevalent worldwide (49.1%), followed by genotypes 3 (17.9%), 4 (16.8%) and 2 (11.0%).^29^ Silva et al. evaluated genotype distribution in a population of more than 1,500 patients living in the states of RS and SC. These data confirmed that genotypes 1 (53.9%) and 3 (40.7%) were most prevalent in southern Brazil, with frequencies that were similar to those found in the present study.^30^

According to the assessment of the geographical distribution of HCV cases, there was higher prevalence of cases in the districts where the populations showed the highest levels of social vulnerability and in a prison where 12 cases (6%) were identified. These districts have low socioeconomic levels and include many places that are used for drug trafficking. Oliveira et al. carried out a geoprocessing analysis on HCV cases in São Paulo and found that HCV infection was associated with low socioeconomic level.^23^ One hypothesis that corroborates the greater presence of HCV cases in regions with low socioeconomic status could be that such individuals lack knowledge about HCV infection routes, such as the sharing of razors, use of pliers, tattooing with non-sterile material, unprotected sexual intercourse and use of needles shared among drug users.^2^^,^^3^^,^^4^^,^^5^^,^^6^

HCV infection and other forms of viral hepatitis are diseases that have an impact on public health worldwide. Although many data suggest that HCV infection could be eliminated over the next 15-20 years, good understanding of HCV infections is still required in order to develop strategies for preventing new infections.^31^ In this regard, support for health promotion and establishment of surveillance, prevention and control measures for these diseases is crucial for improving patients’ quality of life and decreasing disease transmission.

## CONCLUSION

This population of HCV cases that were diagnosed and notified between 2002 and 2015 in the municipality of Santa Cruz do Sul, was characterized by adult individuals aged 41 years and over, who mostly had chronic hepatitis C and were undergoing treatment. The main transmission routes were illicit drugs and blood transfusions. There were high rates of HIV coinfection and abandonment of disease monitoring, and the cases were predominantly in neighborhoods with low socioeconomic status. In this regard, the present study helps to alert the health authorities about the importance of this disease and the need to implement coping strategies, while also encouraging other studies, with the aim of improving the understanding of the HCV infection situation in other locations.
